# Prophylactic Administration of Fucoidan Represses Cancer Metastasis by Inhibiting Vascular Endothelial Growth Factor (VEGF) and Matrix Metalloproteinases (MMPs) in Lewis Tumor-Bearing Mice

**DOI:** 10.3390/md13041882

**Published:** 2015-04-03

**Authors:** Tse-Hung Huang, Yi-Han Chiu, Yi-Lin Chan, Ya-Huang Chiu, Hang Wang, Kuo-Chin Huang, Tsung-Lin Li, Kuang-Hung Hsu, Chang-Jer Wu

**Affiliations:** 1 Department of Traditional Chinese Medicine, Chang Gung Memorial Hospital, Keelung 20401, Taiwan; E-Mail: huangtsehung@gmail.com; 2 Graduate Institute of Clinical Medicine Sciences, Chang Gung University, Taoyuan 33302, Taiwan; 3 Department of Food Science, National Taiwan Ocean University, Keelung 20224, Taiwan; E-Mails: chiuyiham@hotmail.com (Y.-H.C.); p19810222@yahoo.com.tw (Y.-H.C.); sandy72066@hotmail.com (H.W.); 4 Department of Life Science, Chinese Culture University, Taipei 11114, Taiwan; E-Mail: phd.elainechan@gmail.com; 5 Aquatic Technology Laboratories, Agricultural Technology Research Institute, Hsinchu 30093, Taiwan; 6 Institute of Biomedical Nutrition, Hung Kuang University, Taichung 43302, Taiwan; 7 Holistic Education Center, Mackay Medical College, New Taipei City 25245, Taiwan; E-Mail: kchsports@mmc.edu.tw; 8 Genomics Research Center, Academia Sinica, Taipei 11529, Taiwan; E-Mail: tlli@gate.sinica.edu.tw; 9 Laboratory for Epidemiology, Department and graduate institute of health care management, Chang Gung University, Taoyuan 33302, Taiwan; 10 Center of Excellence for the Oceans, National Taiwan Ocean University, Keelung 20224, Taiwan

**Keywords:** fucoidan, sulfated polysaccharide, lung carcinoma, metastasis, cachexia, chemo-preventative agent

## Abstract

Fucoidan, a heparin-like sulfated polysaccharide, is rich in brown algae. It has a wide assortment of protective activities against cancer, for example, induction of hepatocellular carcinoma senescence, induction of human breast and colon carcinoma apoptosis, and impediment of lung cancer cells migration and invasion. However, the anti-metastatic mechanism that fucoidan exploits remains elusive. In this report, we explored the effects of fucoidan on cachectic symptoms, tumor development, lung carcinoma cell spreading and proliferation, as well as expression of metastasis-associated proteins in the Lewis lung carcinoma (LLC) cells-inoculated mice model. We discovered that administration of fucoidan has prophylactic effects on mitigation of cachectic body weight loss and improvement of lung masses in tumor-inoculated mice. These desired effects are attributed to inhibition of LLC spreading and proliferation in lung tissues. Fucoidan also down-regulates expression of matrix metalloproteinases (MMPs), nuclear factor kappa-light-chain-enhancer of activated B cells (NF-κB) and vascular endothelial growth factor (VEGF). Moreover, the tumor-bearing mice supplemented with fucoidan indeed benefit from an ensemble of the chemo-phylacticity. The fact is that fucoidan significantly decreases viability, migration, invasion, and MMPs activities of LLC cells. In summary, fucoidan is suitable to act as a chemo-preventative agent for minimizing cachectic symptoms as well as inhibiting lung carcinoma metastasis through down-regulating metastatic factors VEGF and MMPs.

## 1. Introduction

The international agency for research on cancer (IARC) estimates in 2012 that >14.1 million people were diagnosed with cancer and >8.2 million people died of cancer or cancer-related diseases [1]. Lung cancer accounts for 19.5% of all cancer deaths, the leading cause of cancer death. The non-small-cell lung cancer (NSCLC) takes up approximately 85% out of all lung cancer cases [2]. The five-year survival rate for patients receiving surgical resection, radiation ablation or systemic chemotherapy is as incredibly low as 10%–15%. Recent studies suggested that the primary neoplasm of lung cancer is prone to invade surrounding tissues and then metastasize to distant organs [3]. Metastasis often determines the survival time and the life quality of lung cancer patients [4].

Tumor metastasis of primary tumor cells to distant organs is a multistep process that follows a typical tumor metastatic cascade, such as uncontrolled cell proliferation, tissue remodeling, angiogenesis and invasion [5]. The colonization of tumor cells in secondary organs generally recruits specific sets of proteins at a given time point. Matrix metalloproteinases (MMPs) are known to be closely related to integrity of extracellular matrix (ECM) and basal membrane, of which their disruption is thus correlated to tumor invasiveness. Husmann *et al.* reported that an increase of MMPs in the human osteosarcoma cell model destructs ECM, thus correlating the level of MMPs to tumor metastasis [6]. In NSCLC, tissue inhibitors of metalloproteinases (TIMPs) reported regulate the NSCLC tumor invasion and metastasis [7]. Generally, high expression of MMPs in lung tissues signals a bad prognosis in NSCLC [8].

It has been known that MMPs promote migration of endothelial cells and facilitate formation of new blood vessels. The density of microvessels of tumorigenesis thus reflects patient’s prognosis. The vascular endothelial growth factor (VEGF) is one of major proangiogenic factors [9]. VEGF promotes vascular endothelial growth and mediates vessel permeability, thus facilitating tumor progression and metastatic spread [10]. Chen *et al.* reported that over-expression of VEGF in small-cell lung cancer patients has to do with lymph node metastasis [11]. Liu *et al.* also reported that the levels of VEGF-B and MMP9 in the NSCLC metastatic patients are significantly elevated [12]. High expression of VEGF-A but low expression of both VEGF-B and VEGF-D manifests both poor time to progression (TTP) and overall survival (OS) in NSCLC [13].

The ocean is a gigantic pool of biologically active substances [14,15,16,17]. Fucoidan, a heparin-like sulfated polysaccharide, is abundant in brown seaweeds. Fucoidan is composed of l-fucose as well as other sugars, such as d-xylose, d-galactose, d-mannose, and glucuronic acid [18]. Several studies have reported that fucoidan carries many desired biological effects, such as anticoagulation/antithrombosis [19], anti-inflammation [20], antioxidation [21], anticancer activity [22], and antiviral activity [23]. Specifically, fucoidan is able to induce senescence against hepatocellular carcinoma [24], induce apoptosis of human breast and colon carcinoma [25], as well as prevent migration and invasion of human lung cancer cells [26]. Additionally, fucoidan is able to suppress tumor growth in NSCLC-bearing nude mice [27], prevent tumor-induced angiogenesis in sarcoma 180-bearing mice [28] and induce apoptosis against 4T1 mouse breast cancer cells [29].

The anticancer mechanism of fucoidan remains far from clear. In this report, we wanted to evaluate the inhibition effects of fucoidan on cachectic symptoms, tumor development, lung carcinoma cell spreading/proliferation, as well as expression of metastasis-associated proteins in an LLC cells-inoculated mice model in order to know whether fucoidan is suitable to serve as a prophylactic agent in the prevention of cancer-cell invasion and metastasis. We also wanted to explore the effect of fucoidan on tumor cell viability, wound healing, invasiveness and MMPs activities. Finally, we summarize that fucoidan is an excellent agent capable of improving cachectic symptoms, inhibiting colonization of lung metastasis, and decreasing tumor cell viability by inhibition of MMPs activities and reduction of VEGF expression.

## 2. Results

### 2.1. Fucoidan Mitigates Cachectic Symptoms in LLC-Inoculated C57BL/6 Mice

**Figure 1:**
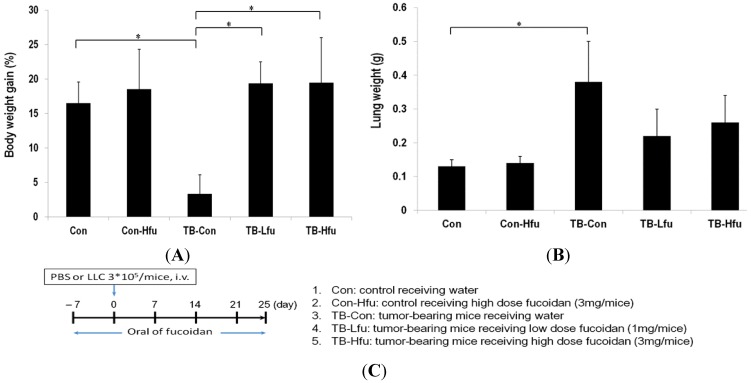
Effects of fucoidan on body weight and lung mass in the LLC xenografted mouse model. At the 25th day, mice were sacrificed and examined for final gains of body weights (**A**) and lung masses (**B**); (**C**) The treatment protocol of fucoidan in tumor-bearing mice. Mice were fed orally with water or low- or high-dose of fucoidan (1 or 3 mg/mice) seven days before tumor implantation. Data are expressed as means ± SD (*n* = 6 mice per group; two independent experiments). Asterisk (*) stands for a significant difference when compared with the control group (*p* < 0.05).

We set out to establish the prophylactic effect of fucoidan by observing cancer cachectic symptoms and tumor development/metastasis, for which the body weight, and Lewis lung carcinoma cell spreading/proliferation were monitored. The alterations of body weight in testing animals are summarized in Figure 1. The body weights are increased by 16.51% ± 3.05% in control mice, while the body weights are slightly increased by 3.34% ± 2.75% in tumor bearing control (TB-Con) mice (Figure 1A), indicating that inoculation of tumor cells results in body weight loss (*p* < 0.05). In contrast, the body weights are significantly increased by 19.35% ± 3.12% and 19.47% ± 6.51% in TB-Lfu and TB-Hfu groups, respectively (*p* < 0.001, Figure 1A).

Table 1 summarizes hematological and spleen immunological parameters. In the TB-Con group, the total red cell (RBC) count is low when compared with those of other groups. The total RBC counts in the mice receiving either a low or high dose of fucoidan are similar to that of control (*p* < 0.05), suggesting that fucoidan can keep tumor-induced RBC steady. In terms of total white cell (WCB) count (including absolute neutrophil, monocyte and lymphocyte), they are all similar. After inoculation of LLC cells, the cell counts of absolute neutrophils, monocytes and lymphocytes were determined to be 6.9 ± 0.8, 2.1 ± 0.6, 0.6 ± 0.1, and 4.4 ± 0.7 × 10^9^/L, respectively; significantly lower than those in control. After administration of fucoidan, the leukopenia effects (including neutropenia and lymphopenia) were considerably reduced (*p* < 0.05; Table 1A), indicating that fucoidan alleviates cachectic leukopenia in tumor-bearing mice. However, the subpopulation distributions of spleenocytes did not change in the group subject to tumor inoculation and fucoidan administration (Table 1B).

The changes of lung masses are relatively minor, which are 0.13 ± 0.02 and 0.14 ± 0.02 g for the control and Con-Hfu mice, respectively, (Figure 1B) as opposed to 0.38 ± 0.12 g for the TB-con mice. These results indicate that fucoidan does not considerably influence the masses of lungs, but LLCs significantly increase lung masses. The increase of lung masses is likely as result of LLCs spreading and proliferation in lung. The lung masses of the TB-Lfu and TB-Hfu groups are 0.22 ± 0.08 and 0.26 ± 0.08 g, respectively, which are significantly smaller than those of the TB-con group (*p* < 0.001, Figure 1B). As a result, the administration of fucoidan does have an effect on reduction of tumor size (Figure 1B).

**Table 1:** Effects of fucoidan on hematological and spleen immunological parameters in the Lewis lung carcinoma (LLC) xenografted mouse model.

Parameter	Con	Con-Hfu	TB-Con	TB-Lfu	TB-Hfu
**(A) Hematology parameter**					
Total red cell count (10^12^/L)	9.7 ± 0.2	9.4 ± 0.2	6.5 ± 0.2	9.2 ± 0.4 ^b^	9.1 ± 0.8 ^b^
Total white cell count (10^9^/L)	18.9 ± 1.4	23 ± 1.0 ^a^	6.9 ± 0.8	14.2 ± 2.0 ^b^	14.2 ± 2.2 ^b^
Absolute neutrophil count (10^9^/L)	3.8 ± 0.7	4.9 ± 0.4 ^a^	2.1 ± 0.6	4.1 ± 0.9 ^b^	3.4 ± 0.1 ^b^
Absolute monocyte count (10^9^/L)	1.1 ± 0.2	1.3 ± 0.1	0.6 ± 0.1	0.9 ± 0.3 ^b^	0.9 ± 0.3 ^b^
Absolute lymphocyte count (10^9^/L)	14.1 ± 0.5	16.8 ± 1.3 ^a^	4.4 ± 0.7	10.7 ± 1.7 ^b^	10.6 ± 1.6 ^b^
**(B) Spleenocyte parameter**					
CD3^+^ (%)	37.6 ± 4.2	35.8 ± 5.6	31.2 ± 2.7	35.5 ± 2.5 ^b^	33.5 ± 1.5
CD4^+^ (%)	19.4 ± 1.8	15.8 ± 0.8 ^a^	16.28 ± 2.6	15.1 ± 2.0	15.4 ± 1.9
CD8^+^ (%)	16.8 ± 3.7	17.3 ± 1.5	14.5 ± 1.7	17.1 ± 3.0	15.4 ± 1.4
CD19^+^ (%)	45.7 ± 5.0	43.4 ± 4.4	47.5 ± 4.4	47.2 ± 3.9	45.4 ± 3.5

Data are expressed as means ± S.E. (*n* = 6). ^a^ *p* < 0.05 *versus* the control group; ^b^ *p* < 0.05 *versus* the LLC cell-inoculated group.

### 2.2. Fucoidan Inhibits Lung Metastatic Colonization of LLC Cells in C57BL/6 Mice

**Figure 2:**
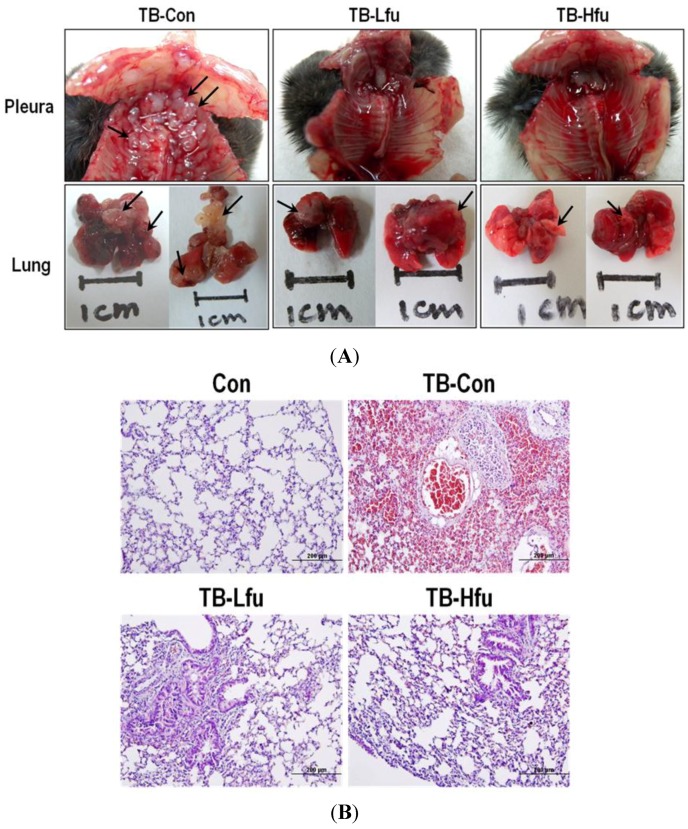
Fucoidan reduces growth of lung tumor in mice. (**A**) Lung, pleural and bronchus tissues. 3 × 10^5^ LLC cells were injected by tail vein in mice. Mice were sacrificed at the 25th day. The solid tumors (indicated by arrows) were spotted on multiple sites in mice; (**B**) Lungs were subjected to histological analysis (H&E stain) for determining metastasis. Six representative samples are shown.

Whether fucoidan is able to inhibit lung metastatic colonization of LLC cells was examined. The LLC cells were injected into C57BL/6 mice via tail vein, and observed tumor formation in lung for 25 days. Pulmonary metastasis of the mice treated with/without fucoidan was assessed by counting the number of tumor nodules on the surface of lungs and pleura under macroscopic or microscopic observation at the 25th day (Figure 2A). Multiple metastatic nodules (with a characteristic opaque tumor spot) appeared in lungs for all TB-Con mice (100%), most of which had tumors across pleural and bronchus surfaces. Interestingly, addition with a low or high dose of fucoidan reduced both the number of metastasis in lungs and impeded the dissemination of tumor cells to adjacent areas (Figure 2A). Histological examination using H&E staining identified LLC colonies in lungs of mice intravenously inoculated with LLC cells (Figure 2B). It is worth noting that the tumor sections of TB-Con mice show noticeable increases of metastatic colonies and tumor cells with mitotic nuclei, which agree with macroscopic observations. The histological analysis confirmed that fucoidan significantly suppresses cancer metastasis to lungs. The low dose treatment of fucoidan reduced the number of lung metastatic foci; the high dose treatment had an even stronger effect (Figure 2B), thus concluding that fucoidan possesses anti-metastatic activity.

### 2.3. Fucoidan Restrains LLCs Metastasis by Suppressing Expression of VEGF and MMPs

**Figure 3:**
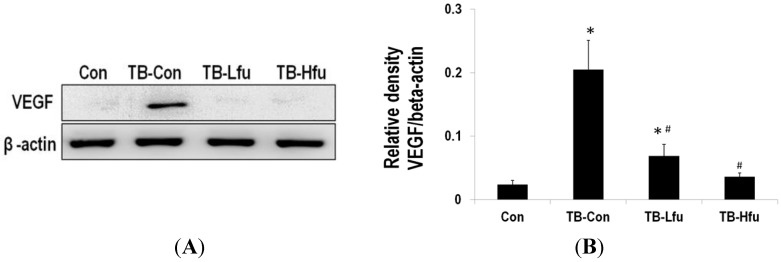
Expression of metastatic proteins in sera of tumor bearing mice treated with fucoidan. (**A**) Western blot analyses of VEGF from representative mice. Expression levels of VEGF normalized to β-actin (**B**). Asterisk (*) indicates a significant difference (*p* value < 0.05) when compared to the con group. Pound (#) indicates a significant difference (*p* value < 0.05) when compared to TB-Con.

**Figure 4:**
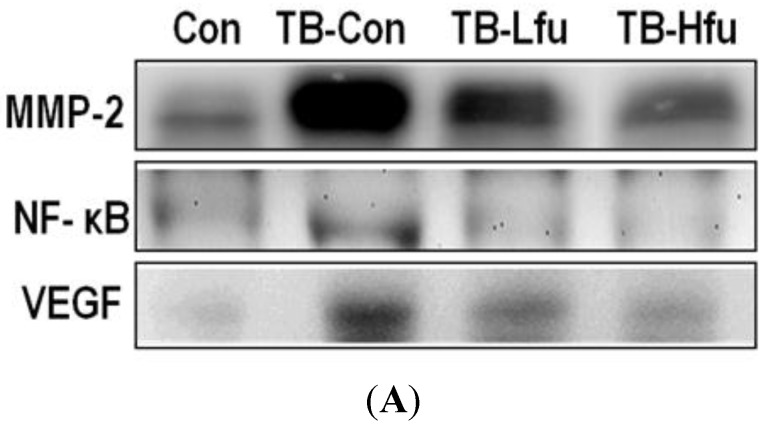

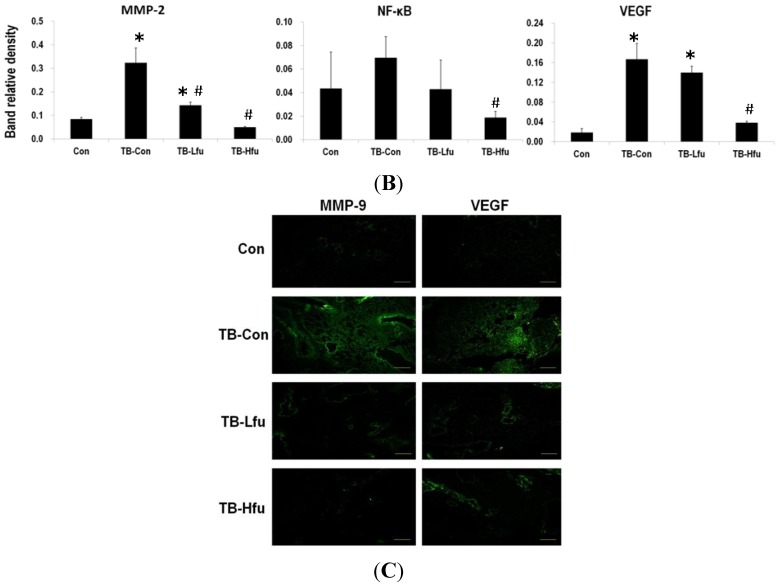
Expression of metastatic proteins in lung tissues of tumor bearing mice treated with fucoidan. (**A**) Western blot analyses of MMP-2, NF-κB and VEGF (from representative samples); (**B**) Protein expression levels that are quantified and expressed as a fold-change relative to the control. Asterisk (*) indicates a significant difference (*p* value < 0.05) when compared to the con group. Pound (#) indicates a significant difference (*p* value < 0.05) when compared to TB-Con; (**C**) Immunofluorescence analysis for lung tumors treated and untreated (TB-Con) with fucoidan. Images are shown at 200× magnification.

To determine the mechanism that fucoidan alleviates lung angiogenesis and metastasis of LLCs, we examined protein expression of VEGF, NF-kB and MMPs in lungs and/or sera. The expression of VEGF in sera and lung tissues are summarized in Figure 3 and Figure 4. The level of VEGF in serum was significantly elevated in the LLCs inoculated mice (Figure 3), which is positively correlated with both the expression level of VEGF in lung tissues (Figure 4) and the severity of tumor metastasis. As shown in the immunohistochemical staining, the VEGF immunoreactivity occurs mainly in the cytoplasm of the lung tissues in TB-Con mice (Figure 4C). Upon the fucoidan treatment (1 mg), the expression of VEGF reduced in sera and cytoplasmas in lung tissues as compared to those in TB-Con. This effect is enhanced when a higher dose (3 mg) of fucoidan was supplemented (Figure 4A,B). MMP-2 and MMP-9 are extracellular metalloproteinases, which play an important role in the degradation of extracellular matrix, thus facilitating cancer cell invasion and migration. It is not surprising that the protein levels of MMP-2 and -9 were increased in mice inoculated with LLCs (Figure 4). Administration of a low or high dose of fucoidan, however, showed declines of MMP-2 and -9. Since the redox-sensitive transcription factor is in charge of sensing oxidative stresses [30], the level of NF-κB is used to index lung cancer progression. The expression of NF-κB in lung tissues is shown in Figure 4, where NF-κB increases significantly upon inoculation with LLCs as compared to that of controls. On receiving either a low or high dose of fucoidan, the expression of NF-κB decreases when compared to that in TB-Con mice. The effect of the high dose is higher than that of the low dose (Figure 4A,B). Fucoidan is thus concluded to carry an anti-metastasis activity.

### 2.4. Fucoidan Has a Cytotoxic Effect on the LLC Cell Line

**Figure 5:**
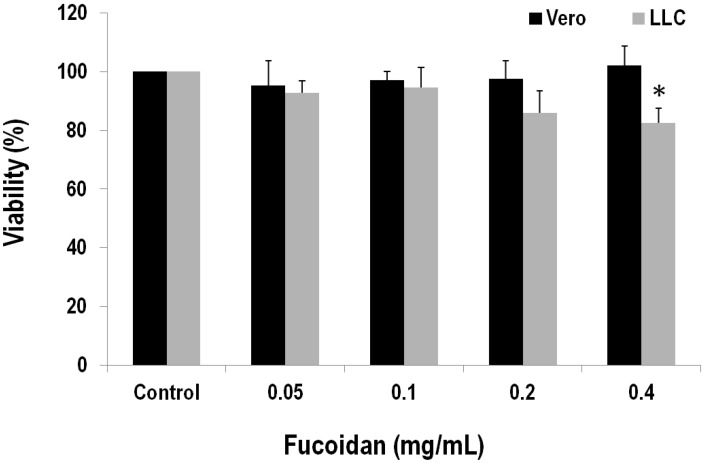
Effects of fucoidan on cell viability in African green monkey kidney Vero and mouse Lewis lung carcinoma cells. Cells were incubated in a culture medium containing various concentrations of fucoidan for 24 h. After the treatment, cell viability was determined by the MTS assay. Values relative to that of vehicle control were determined, in which the cell viability of control is set as 100%. Data (each value is an average of at least three independent experiments (six tests)) are presented as mean ± SEM. Asterisk (*) indicates a significant difference (*p* value < 0.05) relative to the vehicle-treated cells.

Having learned that fucoidan is able to inhibit tumor growth and metastasis in the Lewis lung carcinoma transplantation model, we were keen to know its molecular/cellular mechanism. For this, the Vero normal kidney epithelial cells and Lewis lung carcinoma cells were incubated for 24 h in the presence of various concentrations of fucoidan. The MTS assay showed that fucoidan damages cell viability of LLCs in a concentration-dependent manner. As shown in Figure 5, the cell viabilities are 92.86% ± 3.97%, 94.57% ± 6.77%, 85.99% ± 7.51% and 82.43% ± 5.08% in the presence of 0.05, 0.1, 0.2 and 0.4 mg/mL fucoidan, respectively. LLCs decline significantly in the presence of 0.4 mg/mL fucoidan when compared to the one with no added fucoidan (*p* < 0.05). In contrast, the Vero normal kidney epithelial cells increase (Figure 5); hence fucoidan sensitizes LLC cancer cells but not Vero normal cells.

### 2.5. Fucoidan Prevents Metastasis of Lung Adenocarcinoma Cells

**Figure 6:**
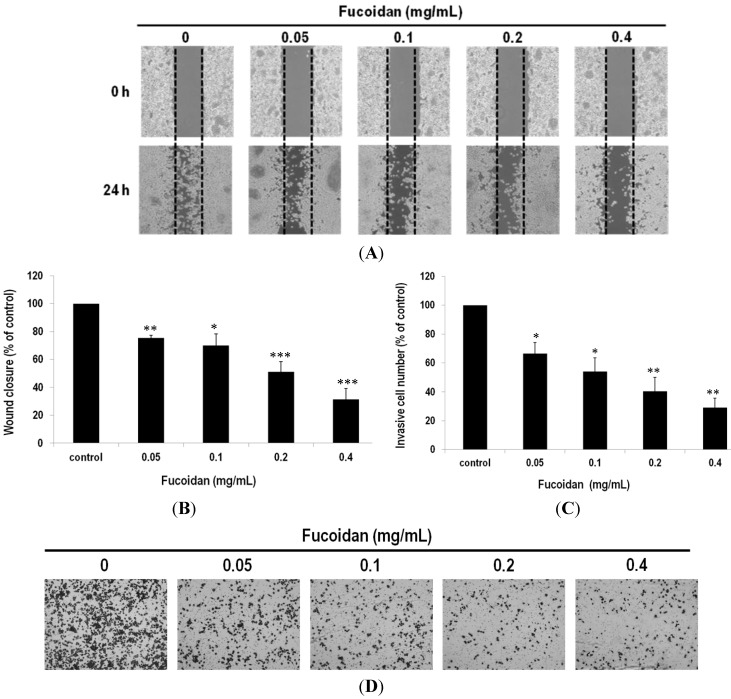
Suppression of migration and invasion of lung adenocarcinoma cells by fucoidan. (**A**) Representative photographs of three independent experiments, showing a dose-dependent inhibition of migration after treatment of fucoidan (24 h). Images of wound closures (10× magnification); (**B**) Black dotted lines indicate the wound edge. The cell-free areas invaded by cells (across the black dotted lines) were quantified by three random fields as shown in the lower panels; (**C**) The invasiveness of the LLC cells were quantified by counting the stained cells that invade into the porous polycarbonate membrane; (**D**) Invasiveness of the LLC cells treated with fucoidan. The LLC cells were pretreated with fucoidan for 24 h and then seeded onto the transwell chamber. Photographs were taken by an inverted microscope with 10× magnification. Data were derived from three independent experiments and presented as mean ± SEM. * *p* < 0.05, ** *p* < 0.001, *** *p* < 0.0001 when compared to the vehicle-treated cells.

To verify the *in vivo* inhibitory effect of fucoidan on LLC metastasis, we tested various concentrations of fucoidan on the migration and invasion of the LLC cells by the wound healing and chamber assays. In the wound-healing assay, the control cells migrated to a wound area where the wound edges became undistinguishable, whereas the cells with addition of fucoidan displayed slower wound closure (Figure 6A). To correlate the cell movement with the quantity of fucoidan, the migration inhibition was determined to be 24.76% ± 2.04%, 29.97% ± 8.15%, 49.03% ± 7.55% and 68.70% ± 7.94% *versus* 0.05, 0.1, 0.2 and 0.4 mg/mL fucoidan, respectively (Figure 6B).

In the invasion chamber assay, matrigel-coated membranes were used to investigate the invasive properties of the cells treated with/without fucoidan. After LLC cells were incubated for 24 h in a transwell assay system, the number of cells for those that had moved through the membrane of the chamber declined as 33.50% ± 7.63%, 45.92% ± 9.35%, 59.61% ± 9.44% and 70.83% ± 6.61% against the number of the control (*p* < 0.01, Figure 6C,D) for the cells added with 0.05, 0.1, 0.2 and 0.4 mg/mL fucoidan, respectively. Fucoidan is thus concluded able to prevent metastasis of lung adenocarcinoma cells.

### 2.6. Effects of Fucoidan on Expression and Activity of MMPs

**Figure 7:**
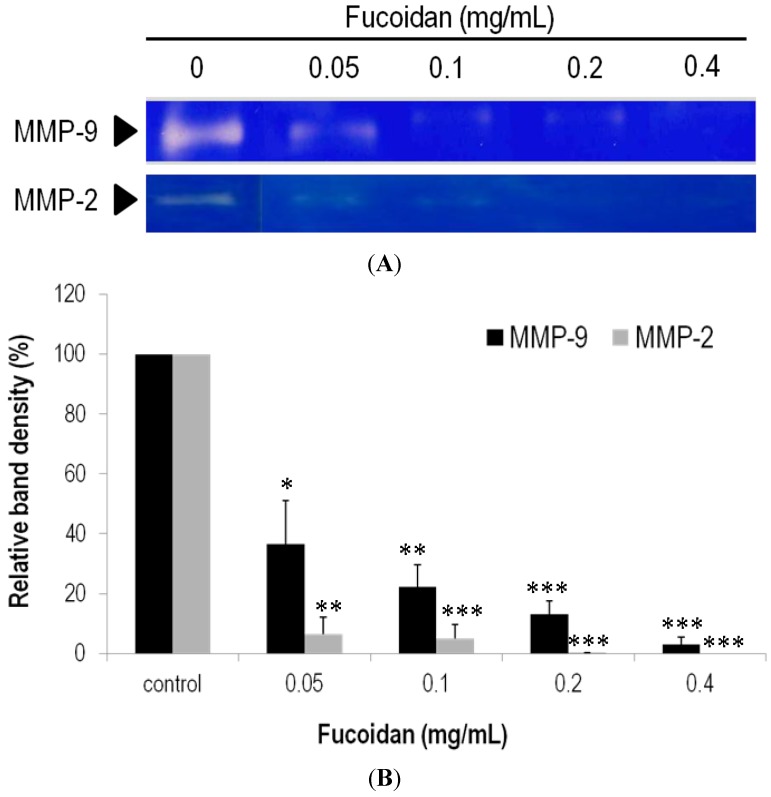
Enzymatic activities of MMP-2 and MMP-9 in the LLC cell lines treated with fucoidan. (**A**) The activity of MMPs was determined by the gelatinase zymography, in which the bright zones stand for gelatin digested; (**B**) The MMPs activity was quantified by measuring the band intensity in the zymography. Data were derived from three independent experiments and presented as mean ± SEM. * *p* < 0.05, ** *p* < 0.001, *** *p* < 0.0001 when compared to the vehicle-treated cells.

Whether the reduced invasion/migration upon addition of fucoidan is related to MMP proteins in LLC cells was examined. As shown in Figure 7A, the enzyme activities of both MMP-2 and MMP-9 declined significantly in the gelatin zymography assays. Namely, the MMP-2 activities dropped to 36.44% ± 14.74%, 22.22% ± 7.30%, 13.07% ± 4.49% and 2.89% ± 2.50% for the samples with 0.05, 0.1, 0.2 and 0.4 mg/mL fucoidan added, respectively (Figure 7B). MMP-9 behaved similarly when exposed to fucoidan. Likewise, the MMP-9 activities dropped to 6.44% ± 5.68%, 5.04% ± 4.59% and 0.12% ± 0.20% for the samples with 0.05, 0.1 and 0.2 mg/mL fucoidan added, respectively (Figure 7B). Interestingly, the MMP-9 activity was not detected when the concentration of fucoidan was raised to 0.4 mg/mL.

## 3. Discussion

Fucoidan was reported with anticancer activity [24,25,26,27,28,29]. It was also reported that the people that consume higher fucoidan-containing seaweeds have low incidences of certain tumors. Given that Lewis lung carcinoma cells (LLCs) specifically invade lungs and that removal of the primary tumor facilitates tumor metastasis, we herein verified that administration of fucoidan considerably reduces the metastatic load in a dose-dependent manner. The fact is partially due to that administration of fucoidan reduces cachectic body weight loss, hematological anemia/leukopenia, and the tumor-induced mass increase of lungs. In all TB-Con animals multiple metastatic nodules located in lungs, where they showed as opaque spots invading bilaterally throughout the pleural and bronchus surfaces. When pretreated with a low or high dose of fucoidan, metastatic nodules declined significantly, strongly supporting that fucoidan has a high anti-metastatic activity.

Metastasis impairs quality of life and results in a poor prognosis [4]. Current mainstay treatments, such as chemotherapy, radiation therapy and target therapy, all come along with severe side-effects. The prophylactic treatment now emerges as a new way to control or prevent micrometastases. An effective anti-VEGF agent ideally is able to block formation of new tumor vessels or even to prune away existing ones. One sad example is that bevacizumab seemed able to wither tumor vessels and reduce tumor microvascular density by 40%–50% in the phase I trial for rectal cancer patients [31], while it failed in the phase III trial as result of increased expression of some angiogenic factors [32]. Prophylactic cranial irradiation has shown a promising outcome, where brain metastases in NSCLC patients were prevented [33]. In our study, fucoidan was shown to prophylactically inhibit lung metastasis colonization of LLC cells in C57BL/6 mice. Fucoidan acts likely to down-regulate the expression of MMPs, NF-κB and VEGF. VEGF was described as a multifunctional angiogenic regulator that stimulates epithelial cell proliferation, blood vessel formation and endothelial cell survival [34]. High levels of VEGF were detected in sera and tumor tissues in mice, well correlating VEGF overexpression with tumor metastasis as well as poor survival rate. Interestingly, fucoidan can reduce the tumor-induced VEGF expression as well as the expression of MMP-2, MMP-9 and NF-κB in lung tissues, suggesting fucoidan restrains cancer cells from invasion and metastasis through suppressing epithelial cell proliferation and blood vessel formation.

To further explore the mechanism underlying fucoidan’s protective effect, we evaluated the effects of fucoidan on cell viability of normal and cancer cells. LLC mouse lung cancer cells are more susceptible to fucoidan than Vero kidney normal cells, as fucoidan significantly reduced viability of LLC cells at the level of 0.4 mg/mL. In contrast, fucoidan does not have cytotoxicity to Vero cells. Our result agrees with previous studies, where fucoidan inhibited the growth of skin and lung cancer cells but enhanced normal cell immune activity [35].

The motility factors, such as MMP-2 and MMP-9, which govern metastasis, have been well documented. On the basis of our results, fucoidan is able to mediate the activities of MMP-2 and MMP-9, also in line with the report that fucoidan suppressed migration and invasion of A549 lung cancer cells by suppressing secretion and/or expression of MMP-2 [26]. Although both LLC and A549 cells are highly invasive and metastatic [36,37], the LLC cells are more susceptible to MMPs under the mediation of fucoidan on the basis of our results.

## 4. Materials and Method

### 4.1. Cells and Cell Culture

Lewis lung carcinoma cells (LLC, C57BL/6 strain mice lung cancer cell line, ATCC CRL-1642) were obtained from the Bioresource Collection and Research Center (Hsinchu, Taiwan). LLC cells were maintained in Dulbecco’s modified Eagle’s medium (DMEM) supplemented with 10% fetal bovine serum. Cells were cultured at 37 °C in a humidified incubator with an atmosphere of 5% CO_2_.

### 4.2. Preparation of Fucoidan Extract

Commercially available fucoidan purified from *F. vesiculosus* (F5631) was purchased from Sigma-Aldrich, Inc. (St. Louis, MO, USA). Due to a substantial difficulty (the fact is that the structures of fucoidan polysaccharides have not yet been determined in detail), the purchased fucoidan was not subjected to further purification. The purchased fucoidan was used to examine its *in vitro* effects on anti-migration and -invasion in a simulated gastric fluid (SGF) or intestinal fluid (SIF) system. The fucoidan powders were dissolved in PBS and the simulated gastric (pH 1.2) and intestinal fluids (pH 7.5) with continuous mixing at 200 rpm for 3 h. The mixture was then placed in an 80 °C water bath (Julabo, Germany) to denature the enzymes in the gastric and intestinal fluids. It was sterilized using a 0.45 mm pore filter (Merck KGaA, Darmstadt, Germany) and stored as “fucoidan extract (20 mg/mL)” at 4 °C until use.

### 4.3. Ethical Approval and Animals

Male C57BL/6 mice (6–8 weeks) were obtained from the National Laboratory Animal Center (Taipei, Taiwan, ROC) and housed in a climate controlled room (12:12 dark-light cycle with a constant room temperature of 21 ± 1 °C). Mice underwent at least 4-day adjustment to new environment and diet before treatments were performed. Food and water were given *ad libitum*. All methods were performed with approval from the Animal Care and Use Committee of National Taiwan Ocean University.

### 4.4. Experimental Design

To examine the effects of fucoidan on the cancer metastasis, mice were divided into five weight-matched groups in the preventive model: (1) control receiving water (Con); (2) control receiving high dose (3 mg/mice) fucoidan (Con-Hfu); (3) tumor-bearing mice receiving water (TB-Con); (4) tumor-bearing mice receiving low dose (1 mg/mice) fucoidan (TB-Lfu); and (5) tumor-bearing mice receiving high dose (3 mg/mice) fucoidan (TB-Hfu). Commercially available fucoidan without further process was used in animal study. One milligram or three milligram fucoidan were diluted in 500 μL water and feed to mice once a day by intragastric gavage 7 days prior to tumor inoculation. At the 7th day post oral administration, 3 × 10^5^ live LLC cells in 100 μL PBS were injected into mice through tail vein. Mice were kept to receive fucoidan or water orally until the due course of the experiment. In all experiments, animals were anaesthetized by CO_2_ inhalation method and weighted at the termination of the experiment on day 25. Following sacrifice, the lung tissues were fixed in 4% paraformaldehyde and stained with hematoxylin and eosin. Final body weight gain was calculated as the difference between the carcass and initial weight.

### 4.5. Blood Sample Analysis

Blood samples (0.5 mL) for measurements of red blood cells (RBC), white blood cells (WBC), lymphocyte, monocyte and neutrophil counts were determined by a blood cell analyzer (Symex K-1000, Sysmex American, Mundelein, IL, USA).

### 4.6. Flow Cytometry

During flow cytometry, at least 5 × 10^5^ spleenocytes were analyzed by Becton–Dickinson FACSan flow cytometer (Franklin Lakes, NJ, USA) with CellQuest software (Becton–Dickinson, Oxford, UK). Lymphocytes were gated based on the expression of CD3 and CD4 or CD8. B cells were gated based on the expression of CD19^+^.

### 4.7. Lewis Lung Carcinoma Cells Metastasis Models

For the passive metastasis model, Lewis lung carcinoma cells (3 × 10^5^ cells in 100 μL PBS) were injected via the tail vein into mice as described previously. At the end of the experiments, the lungs were harvested and the surface nodules were counted to evaluate the metastatic spread of the tumor. Tissues with metastases were either photographed for gross morphology or further analyzed by histology. For pulmonary nodule enumeration, the number of metastatic foci in H&E stained lung sections was counted in a blinded fashion.

### 4.8. Western Blot Analysis

Confluent cell lines cultures were washed with buffered salt solution and treated with fucoidan in the serum-free medium for 24 h. At the end of the experiments, medium was removed and 500 mL was concentrated using Microcon concentrators (Millipore, Bedford, MA, USA) for 30 min at 25 °C. Concentrated samples with equal amounts of protein (25 mg) were mixed with 2 mL reducing sample buffer and resolved by SDS/PAGE, transferred to nitrocellulose membrane (Bio-Rad, Hercules, CA, USA), and the blot was probed with polyclonal goat anti-mice MMP-2, NF-κB, VEGF and β-actin antibodies (Santa Cruz Biotechnology, Santa Cruz, CA, USA). Immunoreactive proteins were visualized by Immobilon™ Western chemiluminescent HRP Substrate kit (Millipore, Bedford, MA, USA). Images were captured and the intensities of the protein bands were analyzed using the Lab works^®^ software (V4.5, UVP Inc., Upland, CA, USA) are expressed as arbitrary optical density unit.

### 4.9. Histopathological Analysis

Tumor and lung tissues were collected from mice, washed carefully by cold normal saline 3 times, then fixed in 10% formalin solution, processed, and embedded in a paraffin film. Sections of 5-μm thick slices of tissues were prepared. The sections were stained with H&E. Microscopic observations were carried out at 200× magnifications.

### 4.10. Immunofluorescence Assay 

Sections were blocked by blocking buffer for 1 h at room temperature then stained with primary antibody at 1:200 dilution for 24 h. The primary antibody was washed by phosphate buffered saline (PBS). Sections were stained with secondary antibody at 1:100 dilution for 24 h at room temperature then washed with PBS. The primary antibodies that were used are as follows: rabbit anti-mouse VEGF (Santa Cruz Biotechnology, Santa Cruz, CA, USA) and rabbit anti-mouse MMP-9 (Santa Cruz Biotechnology, Santa Cruz, CA, USA). The secondary antibodies were FITC-conjugates goat anti-rabbit IgG (Sigma, Sanint Louis, MO, USA).

### 4.11. Cell Viability Assay

(3-(4,5-dimethylthiazol-2-yl)-5-(3-carboxymethoxyphenyl)-2-(4-sulfophenyl)-2H-tetrazolium) (MTS) assay is a colorimetric assay based on the ability of viable cells to change from soluble yellow tetrazolium salt to blue formazan crystals. LLC cells (0.5 × 10^4^ cells/mL) were first pretreated with varying concentrations of fucoidan for 24 h. After drugs treatment, cells were washed with incubation buffer, collected by centrifugation, and then suspended in the incubation buffer, containing 0.5 mg/mL MTS for 4 h. After MTS treatment, cells were collected by centrifugation, and then suspended in DMSO for 10 min to thoroughly dissolve the dark blue crystals. The absorbency at a wavelength of 490 nm was measured by spectrophotometer. The cell viability was determined by comparing the results with the absorbency of the untreated cells.

### 4.12. Cell Migration and Invasion Assays

LLC cells migration and invasion were determined using the wound healing assays and transwell plate as previously described. Briefly, for wound healing assays, LLC cells (5 × 10^5^/well) were seeded and grown overnight to 90%–95% confluence in 12-well plates before wounds of similar size were introduced into the monolayer by a sterile pipette tip. The monolayers were rinsed with phosphate buffer saline (PBS) to remove detached cells and then cultured in medium containing varying concentration of fucoidan. The speed of wound closure was documented 24 h post-wounding using the Nikon Eclipse TE2000U microscope (Melville, NY, USA).

Cell invasion assays were performed using transwell cell culture inserts (Becton Dickinson, Franklin Lakes, NJ, USA). As many as 3.5 × 10^5^cells were placed in the top part of the chamber. The top part of the chamber was filled with DMEM or with medium supplemented with varying concentration of fucoidan, while DMEM supplemented with 10% FBS was added in the bottom part of the chamber. Incubation was carried out at 37 °C for 24 h. The filters were removed and fixed with 100% methanol for 8–10 min at room temperature. Cells on the upper filter surface were removed with a cotton swab. The filters were stained with 0.2% w/v crystal violet, washed with PBS (pH 7.4), and observed under a light microscope operating at 200× magnification. The invasion index was defined as the ratio of the percent invasion obtained with invaded cells (LLC cells) to the percent invaded obtained with non-invaded cells.

### 4.13. Gelatin Zymography

LLC cell lines were starved for 24 h with medium containing no FBS. Subsequently, the cells in media containing 0.5% FBS were stimulated with varying concentration of fucoidan for different time periods, and then the supernatants were collected. The samples were analyzed with gelatin zymography, (0.1% w/v) gelatin (Sigma, Sanint Louis, MO, USA) as the substrate. Each lane was loaded with a total protein concentration of 3 μg and subjected to SDS-PAGE electrophoresis at 48 °C. Gels were washed twice in 50 mM Tris (pH 7.4) containing 2.5% (v/v) Triton X-100 for 1 h, followed by two 10-min rinses in 50 mM Tris (pH 7.4). After SDS removal, the gels were incubated overnight in 50 mM Tris (pH 7.5) containing 10 mM CaCl_2_, 0.15 M NaCl, 0.1% (v/v) Triton X-100, and 0.02% sodium azide at 37 °C under constant gentle shaking. After incubation, the gels were stained with 0.25% Coomassie brilliant blue R-250 (Sigma, Sanint Louis, MO, USA) and destained in 7.5% acetic acid with 20% methanol. The gelatinase bands appeared white on a blue background.

### 4.14. Statistical Analysis

All experiments were performed at least 3 times, each time in triplicate. Data were analyzed by multivariate ANOVA test. If a significant difference was found, a least significant differences (LSD) multiple comparison test was used to identify significant groups. Statistical analyses used The Statistical Software Package for the Social Sciences, version 12.0.1 for Windows (SPSS Inc., Chicago, IL, USA). A *p* value < 0.05 was considered statistically significant.

## 5. Conclusions

Fucoidan exhibits an anti-metastasis activity, which could ensure traditional therapeutic efficacy. The beneficial effects of fucoidan are largely attributed to down-regulation of MMPs, NF-κB and VEGF.
